# Analgesic and side effects of intravenous recombinant Phα1β

**DOI:** 10.1590/1678-9199-JVATITD-2019-0070

**Published:** 2020-04-17

**Authors:** Flavia Karine Rigo, Mateus Fortes Rossato, Vanessa Borges, Juliana Figueira da Silva, Elizete Maria Rita Pereira, Ricardo Andrez Machado de Ávila, Gabriela Trevisan, Duana Carvalho dos Santos, Danuza Montijo Diniz, Marco Aurélio Romano Silva, Célio José de Castro, Thiago Mattar Cunha, Juliano Ferreira, Marcus Vinicius Gomez

**Affiliations:** 1 Graduate Program in Health Sciences, University of the Extreme South of Santa Catarina (UNESC), Criciúma, SC, Brazil.; 2 Department of Pharmacology, Ribeirão Preto Medical School, University of São Paulo (USP), Ribeirão Preto, SP, Brazil.; 3 Institute of Education and Research of Santa Casa Belo Horizonte, Santa Casa of Belo Horizonte Group, Belo Horizonte, MG, Brazil.; 4 Department of Neurosciences, School of Medicine, Federal University of Minas Gerais (UFMG), Belo Horizonte, MG, Brazil.; 5 Department of Pharmacology, Federal University of Santa Catarina, Florianópolis, SC, Brazil.

**Keywords:** Recombinant Phα1β, Analgesia, Neuropathic pain, Intravenous drug delivery system, Side effects, Cardiac function, Motor activity, Biochemicals

## Abstract

**Background:**

Intrathecal injection of voltage-sensitive calcium channel blocker peptide toxins exerts analgesic effect in several animal models of pain. Upon intrathecal administration, recombinant Phα1β exerts the same analgesic effects as the those of the native toxin. However, from a clinical perspective, the intrathecal administration limits the use of anesthetic drugs in patients. Therefore, this study aimed to investigate the possible antinociceptive effect of intravenous recombinant Phα1β in rat models of neuropathic pain, as well as its side effects on motor, cardiac (heart rate and blood pressure), and biochemical parameters.

**Methods:**

Male Wistar rats and male Balb-C mice were used in this study. Giotto Biotech® synthesized the recombinant version of Phα1β using *Escherichia coli* expression. In rats, neuropathic pain was induced by chronic constriction of the sciatic nerve and paclitaxel-induced acute and chronic pain. Mechanical sensitivity was evaluated using von Frey filaments. A radiotelemeter transmitter (TA11PA-C10; Data Sciences, St. Paul, MN, USA) was placed on the left carotid of mice for investigation of cardiovascular side effects. Locomotor activity data were evaluated using the open-field paradigm, and serum CKMB, TGO, TGP, LDH, lactate, creatinine, and urea levels were examined.

**Results:**

Intravenous administration of recombinant Phα1β toxin induced analgesia for up to 4 h, with ED_50_ of 0.02 (0.01-0.03) mg/kg, and reached the maximal effect (E_max_ = 100% antinociception) at a dose of 0.2 mg/kg. No significant changes were observed in any of the evaluated motor, cardiac or biochemical parameters.

**Conclusion:**

Our data suggest that intravenous administration of recombinant Phα1β may be feasible for drug-induced analgesia, without causing any severe side effects.

## Background

Pain, one of the most common symptoms in human diseases, remains one of the most poorly understood sensations, with insufficient clinical management despite the large number of pain-relieving drugs available on the market. To overcome this problem, numerous studies have been conducted to elucidate neuronal pathways of pain and develop new treatments [1]. A considerable number of research studies have focused on N-type calcium channel inhibitors to develop novel analgesic drugs [2]. The N-type calcium channel is most known for its role in neurotransmission in sensory neurons. Neuronal voltage-sensitive calcium channels (VSCCs) are expressed mainly at the presynaptic nerve terminals; they allow calcium influx and depolarization-induced neurotransmitter release from both central and peripheral nerves [3, 4]. To date, most drugs that target VSCCs are peptide toxins isolated from venomous animals. The synthetic peptidyl toxin ziconotide (ω-conotoxin MVIIA) is a potent and selective blocker of N-type calcium channel [5]. It has been reported as a promising therapy for peripheral neuropathy [6]. Ziconotide is mainly used via intrathecal (i.t.) administration due to the expected systemic toxicity of ω-conotoxin [7]. Although i.t. ziconotide reduces pain and improves the quality of life of patients with neuropathic pain, its therapeutic window is narrowed with by severe, dose-limiting side effects [8-11]. Intravenous (i.v.) administration of ziconotide exerts no antihyperalgesic effect in rats with diabetic neuropathic pain [12]. There remains a critical need to identify N-type VGCC inhibitors with improved safety and alternative delivery methods for the management of neuropathic pain [13]. 

Our group has extensively described different toxins isolated from purified venom of the spider *Phoneutria nigriventer*, such as PnTx3-6. This toxin was patented under the name Phα1β. It causes blockage of the mammalian VSCCs expressed in HEK cells, leaving LVA (T-type Ca^2+^ channel) unaffected [14]. Phα1β (i.t.) induces antinociception with a higher therapeutic window than that of ω-conotoxin MVIIA (ziconotide) [10]. In addition, i.t. Phα1β has exhibited marked analgesic effect without toxicity in relevant rodent models of pain [15-17], thus showing great potential as a new analgesic drug/therapy for neuropathic pain [10]. However, the widespread use of i.t. Phα1β as an analgesic drug is limited owing to the requirements for this route of administration. It would be more practical and affordable to develop a new safe and efficient analgesia method via a parenteral route. Thus, the present study aimed to investigate the analgesic and side effects of intravenous (i.v.) recombinant Phα1β toxin in rodent models of pain.

## Materials and Methods

### Animals

In this study, we used 72 adult male Wistar rats (180 g, 6 weeks old) divided into 12 groups (n = 6) and 30 adult male Balb-C mice (20 g, 6 weeks old) divided into five groups (n = 6). All animals were acclimatized in the laboratory for at least 2 h before testing. They were used only once throughout the experiments. While in the home cage environment, the animals were allowed free access to water and food. The room temperature was maintained at 22 ± 1°C, and the room illumination was on a 12/12-hour light/dark cycle. The experiments were carried out following the current guidelines for the care of laboratory animals, and adhered to the ethical guidelines for the investigations of experiments on conscious animals [18]. The present study was approved by the Ethics Committee of the Federal University of Minas Gerais, Brazil, under protocol n. 347/2012. Moreover, the number of animals and the intensity of the noxious stimuli used were the minimum levels necessary to manifest the consistent effects of the drug treatments. All experiments were performed in a single-blind manner to avoid possible observer bias results. Behavioral observations were performed before the induction of neuropathy (basal), after initiation of neuropathy (time zero), and 15 to 360 min after drug treatment.

### Drugs and Treatment

Giotto Biotech® synthesized the recombinant version of Phα1β via *Escherichia coli* expression [19]. The peptide molecular weight (Mw) was 6045 kDa and the sequence of the 55 amino acids was ACIPRGEICTDDCECCGCDNQCYCPPGSSLGIFKCSCAHANKYFCNRKKEKCKKA. The purity of the recombinant toxin is higher than 90% by SDS PAGE. The stock solution was prepared in phosphate-buffered saline (PBS; 137 mM NaCl, 2.7 mM KCl, and 10 mM phosphate buffer) and stored in siliconized plastic tubes at -20ºC. The solutions were diluted to the desired concentration just before use. 

Next 50 μL of recombinant Phα1β, morphine (positive control for antinociceptive effect), or PBS was administered intravenously with a needle (30 G) into the caudal veins of the rats [20]. The animals’ tails were submerged into a water bath at 40ºC for 5 s before the i.v. injection. 

### Mechanical Hyperalgesia - von Frey Test

Mechanical sensitivity in rats was evaluated by using von Frey filaments and the up-down paradigm [21, 22]. After acclimatization (1 h) in individual clear Plexiglas boxes with wire mesh floor (9 × 7 × 11 cm), von Frey filaments of increasing stiffness (0.02-10 g) were applied to the hind paw plantar surfaces of the rats for no more than 3 s with sufficient pressure to bend the filament. Upon the absence of response (paw lifting), the next filament with an increased weight was applied, whereas a response to the next weaker filament was applied until six stimulations of four consecutive positive/negative responses were performed. The mechanical threshold was expressed as *log* in milligrams (mg) and was evaluated several times after i.v. administration of the recombinant Phα1β, morphine, or PBS. 

### Chronic Constriction of the Sciatic Nerve

A chronic constriction injury (CCI) of the sciatic nerve was examined by using a previously described method, with minor modifications [23]. Rats were anaesthetized with 90 mg/kg ketamine plus 3 mg/kg of xylazine intraperitoneally (i.p). The sciatic nerve was exposed, and a partial ligation was made by tying one-third to one-half of the dorsal portion of the nerve. Sham-operated rats were anaesthetized, and the nerves were exposed, but no ligation was performed. Mechanical sensitivity was measured before and at 7 days after surgery in a single-blind manner to avoid possible observer bias [24].

### Paclitaxel-Induced Acute and Chronic Pain

To evaluate paclitaxel-induced pain syndrome, rats were initially divided into two groups. The first group received a single injection of paclitaxel (1 mg/kg, i.p) to induce acute painful syndrome, and the mechanical sensitivity was measured before and at 24 h after injection. The second group received four consecutive paclitaxel injections (1 mg/kg, i.p) on alternate days. The acute pain was evaluated before and at 15 days after the first of the four paclitaxel injections [25]. 

### Adverse Effect Assessments

The mice were anaesthetized with isoflurane (2% for induction and 1% for maintenance). The left carotid was exposed for implantation of a radio telemeter transmitter (TA11PA-C10; Data Sciences, St. Paul, MN, USA). Next, behavioral and cardiovascular side effects were analyzed. Seven days after the surgical procedure, the mice were treated with the recombinant Phα1β toxin (0.2, 0.6, or 1.8 mg/kg, i.v.). Locomotor activity (cpm), mean arterial pressure (mmHg), and heart rate (beats per minute, bpm) were continuously recorded for 24 h after treatment. Serpentine-like tail movements, body shaking, and dynamic allodynia were also evaluated using a 7-point scale, as previously described [26, 27]. Cardiac activity was measured by implanting a telemeter transmitter at the left carotid, and an open-field test was conducted using a fully computerized measurement system, with crossing and rearing as parameters

### Open-field Test

To reinforce and confirm the locomotor data from animals in the box, locomotor activity was also evaluated using the open-field paradigm. Briefly, at 2 h after treatment with vehicle, morphine (20 mg/kg, i.v., positive control for pain), or recombinant Phα1β (0.2, 0.6, or 1.8 mg/kg, i.v.), mice were introduced to individual boxes (50 × 50 × 25 cm) to which they had not been previously exposed. Spontaneous activities were measured [28] for over 5 min by the number of crossings (horizontal movements) and the number of rearing (vertical) movements. The latency time to escape the central square, which reflects the anxiolytic/anxiogenic effect, was also measured [28].

### Evaluation of biochemical parameters

After 24 h of recombinant Phα1β treatment (0.2, 0.6, or 1.8 mg/kg, i.v.), the mice were sacrificed. Blood samples were collected, and serum was isolated and frozen (-10ºC) until analysis. Serum CKMB, TGO, TGP, LDH, lactate, creatinine, and urea levels were evaluated using Bioclin^®^ colorimetric assay according to the manufacturer's instructions. 

### Statistical Analyses

Statistical analyses were performed using the software Graph Pad Prism, Version 5.01. The data were analyzed using one-way or two-way analyses of variance followed by the Bonferroni or Student-Newman-Keuls test, when appropriate. Adverse effects were assessed by the Kruskal-Wallis test followed by Dunn's test, when applicable. For analyses of proportions, the χ^2^test was used. The 50% effective dose (ED_50_) values were calculated by nonlinear regression. These values were attained by using a dose-response equation that was adjusted to provide the best description of the values obtained from the individual experiments. Values of *p*< 0.05 were considered significant. The results are presented as mean ± standard error (mechanical threshold - *log* mg), median ± interquartile range (adverse effect scores), or geometric means accompanied by their respective 95% confidence limits (for ED_50_).

## Results

**Figure 1.**
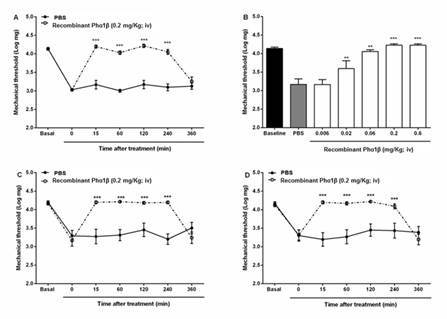
Effects of intravenous (i.v.) recombinant Phα1β administration in rats with chronic pain. **(A)** Time-course and **(B)** dose-response effect of recombinant Phα1β treatment on CCI-induced mechanical allodynia; and time course of recombinant Phα1β (0.2 mg/kg i.v.) in rats with paclitaxel-induced **(C)** acute or **(D)** chronic pain. The results are expressed as mechanical threshold (*log* mg), as measured by von Frey filaments. Mean and SEM values are represented by point and vertical line, respectively (12 groups with 6 rats per group, 72 rats in total). **p < 0.01 and ***p < 0.001 represent significant differences between recombinant Phα1β and PBS treatments, as analyzed by (**B**) one-way or (**A**, **C** and **D**) two-way analysis of variance followed by (**B**) Dunnett’s or (**A**) Bonferroni’s *post-hoc* test (n = 3 to 5).

Intravenous (i.v.) administration of recombinant Phα1β toxin at 0.2 mg/kg significantly reduced CCI-induced mechanical hypersensitivity of neuropathic nociception. The analgesic effects of the recombinant Phα1β lasted for 4 h (Figure 1A), reaching 67.7 ± 9.1% inhibition at a dose of 0.06 mg/kg (i.v.) and 100% inhibition (I_max_ = 100%) at a dose of 0.2 mg/kg (i.v.), with an ED_50_ of 0.02 (0.01- 0.03) mg/kg (Figure 1B). Administration of i.v. recombinant Phα1β toxin caused no changes in the mechanical threshold of sham-operated rats (data not shown). Once the analgesic effect of i.v. recombinant Phα1β on CCI was characterized, we evaluated its effect on paclitaxel-induced hypersensitivity. Mechanical hypersensitivity was measured at 24 h after a single paclitaxel injection (1 mg/kg; i.p - acute painful syndrome) or 7 days after for alternating paclitaxel injections (1 mg/kg; i.p - chronic neuropathic syndrome). Intravenous administration of 0.2 mg/kg recombinant Phα1β inhibited both acute (Figure 1C) and chronic (Figure 1D) mechanical hyperalgesia for up to 4 h post-administration with 100% inhibition (I_max_ = 100%). 

**Figure 2.**
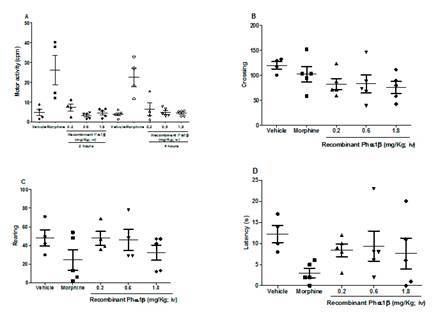
Effects of intravenous (i.v.) recombinant Phα1β injection on mouse motor function. **(A)** Spontaneous motor activity, **(B)** crossing, **(C)** rearing, and **(D)** latency to enter the central square in the open-field test. The individual behavioral data plotted as scatter plots and the means as a horizontal black line for 5 groups with 6 mice per group, 30 mice in total. Morphine increased motor activity at 2 and 4 h after administration, ***p < 0.001 (n = 4 to 5).

To evaluate whether i.v. administration of Phα1β recombinant toxin induces the development of acute toxicities, we evaluated some possible side effects that had been predicted for the calcium channel blockers when administered by this route. Thus, Balb-C mice treated with morphine (20 mg/kg i.v. - positive control) or different doses recombinant Phα1β (0.2, 0.6 or 1.8 mg/kg i.v.) were observed at different time points after each treatment. Morphine increased motor activity at 2 and 4 h after administration, and tended to decrease the number of rearing-behavior instances and the latency time to escape the central square in the open-field test, showing motor changes and anxiety behavior (Figure 2). In addition, no significant difference or change was observed in mice treated with recombinant Phα1β (0.2, 0.6, or 1.8 mg/kg i.v.). No change was observed in the serpentine-like tail movements, body shaking or allodynia at any time-point.

**Figure 3.**
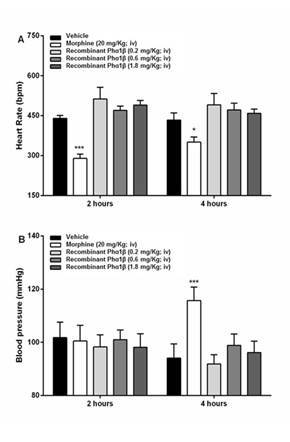
Effects of intravenous (i.v.) recombinant Phα1β administration on mouse cardiac function. **(A)** Heart rate and **(B)** blood pressure measured at 2 and 4 h after vehicle, morphine (20 mg/kg i.v.), or recombinant-Phα1β (0.2, 0.6, or 1.8 mg/ kg i.v.) treatment. The mean and SEM are represented by each bar and vertical line, respectively (5 groups with 6 mice per group, 30 mice in total). *p < 0.05 and ***p < 0.001 represent significant differences between Phα1β recombinant and PBS treatments, as analyzed by one-way analysis of variance followed by Dunnett’s *post-hoc* test (n= 4 to 5).

Similarly, morphine also induced significant changes in heart rate at 2 and 4 h after administration, as well as a 14 mmHg increase in blood pressure at 4 h after administration; these events were not observed in mice treated with recombinant Phα1β toxin (0.2, 0.6, or 1.8 mg/kg i.v.) (Figure 3 and Table 1). Morphine also elevated locomotor activity. Decreased heart rate and increased locomotor activity may directly affect blood pressure, as shown by the peak blood pressure observed at 4 h after morphine treatment. No effects on these parameters were produced by the recombinant Phα1β treatment (0.2, 0.6, or 1.8 mg/kg i.v.). At 24 h after treatment, the mice were sacrificed, and serum samples were collected. No significant changes in CKMB, TGO, TGP, LDH, lactate, urea or creatinine levels were observed after treatment with vehicle, morphine, or recombinant Phα1β (0.2, 0.6, or 1.8 mg/kg i.v.) (Table 2). There were no histological alterations in the liver, heart or kidney tissue after i.v. administration of recombinant Phα1β toxin, thus confirming the safety of the drug.

**Table 1.** Full time course of side effects evaluated after an intravenous administration of vehicle, morphine or recombinant Phα1β in the activity box

Time (h)	Adverse effect								
	Activity (cpm)			Blood pressure (mmHg)			Heart rate (bpm)		
	Vehicle	Morphine	Toxin	Vehicle	Morphine	Toxin	Vehicle	Morphine	Toxin
Basal	5.3 ± 1.0	5.4 ± 1.7	6.3 ± 1.5	110.0 ± 5.7	97.4 ± 3.8	87.0 ± 1.0	443.1 ± 25.9	483.6 ± 27.2	442.5 ± 25.4
2	4.7 ± 1.5	26.2 ± 6.7*	7.2 ± 2.6	101.7 ± 5.8	100.5 ± 5.9	98.2 ± 4.1	440.0 ± 10.0	292.8 ± 12.7*	512.8 ± 39.0
4	3.8 ± 0.8	22.4 ± 4.2*	6.4 ± 1.8	94.0 ± 5.3	115.7 ± 5.1*	91.9 ± 3.1	433.6 ± 23.6	331.6 ± 15.6*	491.1 ± 37.9
6	16.5 ± 5.6	7.9 ± 2.4	16.1 ± 3.3	106.2 ± 8.5	107.9 ± 3.0	96.9 ± 4.7	459.7 ± 18.7	547.5 ± 18.5	528.3 ± 41.9
8	18.3 ± 6.5	3.8 ± 1.1	16.6 ± 4.8	111.8 ± 4.3	102.9 ± 3.0	102.0 ± 2.1	540.4 ± 23.3	505.9 ± 11.6	568.5 ± 24.0
10	17.5 ± 9.8	3.9 ± 1.1	14.7 ± 4.7	105.3 ± 6.1	100.0 ± 4.0	105.3 ± 3.3	492.4 ± 19.8	475.2 ± 10.9	580.5 ± 36.9
12	11.0 ± 2.0	4.7 ± 1.1	5.0 ± 0.5	107.3 ± 4.5	100.6 ± 5.9	94.0 ± 2.4	501.0 ± 32.6	467.4 ± 19.5	470.9 ± 23.5
14	3.0 ± 1.6	4.3 ± 0.5	4.7 ± 1.2	96.0 ± 5.3	93.5 ± 4.0	92.0 ± 2.1	438.2 ± 28.1	420.7 ± 16.9	460.8 ± 18.5
16	2.7 ± 0.6	4.0 ± 1.4	4.3 ± 2.6	100.8 ± 8.9	95.4 ± 3.7	87.6 ± 1.5	456.6 ± 36.0	407.7 ± 19.9	425.9 ± 26.4
18	4.0 ± 1.5	1.5 ± 1.3	2.8 ± 0.4	95.3 ± 6.4	89.2 ± 2.3	86.7 ± 2.6	405.6 ± 24.1	371.2 ± 12.3	417.4 ± 32.2
20	2.9 ± 0.5	3.7 ± 2.9	4.3 ± 1.2	91.7 ± 3.6	96.4 ± 5.2	97.2 ±5.4	432.8 ± 15.2	381.9 ± 22.2	499.8 ± 53.2
22	3.0 ± 0.7	8.7 ± 1.8	5.2 ± 1.6	92.4 ± 3.1	102.0 ± 3.6	90.7 ± 3.1	416.5 ± 11.1	482.8 ± 32.2	448.7 ± 30.4
24	2.9 ± 0.7	4.7 ± 1.8	4.6 ± 1.7	92.4 ± 2.9	97.8 ± 1.9	89.5 ± 2.8	416.7 ± 20.4	434.9 ± 24.8	446.4 ± 33.8

*p < 0.05 represents statically significant differences between groups treated with vehicle, morphine (20 mg/kg i.v.) or recombinant Phα1β (0.2 mg/kg i.v.), according two-way analysis of variance (ANOVA) followed by Bonferroni’s post-test. (h = hour; cpm = counts per minute; bpm = beats per minute; N.D. = not detected) (4 groups of 8 Balb-C mice - total 32 mice).

**Table 2.** Biochemical parameters evaluated in serum samples 24 hours after vehicle, morphine or recombinant Phα1β administrations

	Treatment				
	Vehicle	Morphine	Recombinant toxin (mg/kg i.v.)		
			0.2	0.6	1.8
CK-MB	73.8 ± 7.6	75.1 ± 9.0	65.7 ± 10.7	75.3 ± 9.0	70.4 ± 9.3
TGO	57.1 ± 2.3	47.4 ± 5.0	53.7 ± 3.9	54.2 ± 4.8	55.8 ± 4.4
TGP	25.0 ± 2.3	19.6 ± 1.8	21.3 ± 1.7	22.1 ± 2.1	21.7 ± 2.4
Urea	72.4 ± 3.8	61.6 ± 2.0	72.2 ± 6.1	69.4 ± 1.2	69.7 ± 2.3
Creatinine	3.7 ± 2.6	3.4 ± 0.1	3.7 ± 0.2	3.6 ± 0.3	5.0 ± 1.6
Lactate	46.9 ± 1.7	41.4 ± 1.4	42.9 ± 1.6	42.0 ± 2.0	40.4 ± 1.8
LDH	637.7 ± 63.8	632.0 ± 62.8	610.5 ± 55.1	624.9 ± 54.2	574.6 ± 60.1

No statistical significant difference was observed between groups treated with vehicle, morphine (20 mg/kg i.v.) or recombinant Phα1β (0.2 mg/kg i.v.).

## Discussion

Administration of an analgesic drug by the i.t. route is invasive and often painful; moreover, it poses a risk of infection and requires surgical skills and expensive specialized equipment. Thus, it is preferable to use this route as a last resort, giving preference to other routes, such as intravenous administration. In the present study, we described the effectiveness of recombinant Phα1β administration via i.v. injection in two distinct animal models of neuropathic pain. It is currently unknown whether Phα1β or its recombinant form can penetrate the blood-brain barrier to reach the spinal VSCCs, or whether Phα1β can target calcium channels outside the central nervous system (CNS). The effect of Phα1β outside the CNS would open the way for its use in accessing VSCC blockers, thereby helping patients that cannot tolerate an intrathecal catheter. Future studies that investigate the mechanism and factors, such as bioavailability, distribution, and tissue-specific effects, of the i.v. administration of the peptide are necessary.

Phα1β (0.2 mg/kg) administration via the i.v. route caused pronounced analgesia (anti-hypersensitivity) in two distinct rat models: chronic constriction injury and the acute and chronic paclitaxel pain models. The native Phα1β is one of the high-voltage calcium channel blockers (HVCCs), whose functional role in neuropathic pain mechanisms has been described [29]. Currently, neuropathic pain management is unsatisfactory and remains a challenge in clinical practice. Neuropathic pain is considered one of the most challenging types of pain to manage with conventional analgesics. Most patients complain of pain at the early or later stages of paclitaxel treatment [25, 30-32]. Paclitaxel-associated acute pain syndrome is a severe and debilitating condition that has been reported in up to 58% of patients [33]. However, no standard therapy has been established for managing paclitaxel-induced acute or chronic neuropathic pain. Previously, we have shown that both PAC-induced acute and chronic pain syndrome can be reduced by i.t. administration of HVCCs blockers, such as Phα1β and ω-conotoxin MVIIA [16]. Both toxins provoke an overall similar antinociceptive effect, despite the fact that Phα1β exhibits fewer side effects than ω-conotoxin MVIIA, thus presenting a better analgesic profile [16]. These effects may be attributable to several factors, including the interaction of Phα1β with other targets, such as different HVCCs P/Q, L-, and R-type channels [14] and TRP cation channels. Phα1β is a selective TRPA1 receptor antagonist [34], a cation channel responsible for detecting and transmitting different types of noxious stimuli, both peripherally and centrally. TRPA1 receptor blockers are well known for producing effective antinociception with reduced side effects. Several studies have highlighted the involvement of HVCCs in chronic neuropathic pain induced by paclitaxel. The N-type VGCC blocker NMED-126 reduces chronic neuropathic pain in rats induced by paclitaxel [35]. We have shown that i.v. administration of Phα1β recombinant toxin caused efficient analgesic effect in rats suffering from nerve-injury-induced neuropathic pain and paclitaxel-induced acute and chronic pain, without causing any cardiac or motor side effects. Therefore, this may constitute a safe alternative for treating chronic pain. 

Ziconotide, either as a monotherapy or in combination with other i.t. drugs, is a potential therapeutic option for patients with refractory neuropathic pain [36]. However, i.t. ziconotide has a narrow therapeutic window [10], produces serious side effects at analgesic doses [8-11], and increases creatine kinase levels [37, 38], which were not observed with i.v. Phα1β. An i.v. injection of 0.2 mg/kg ziconotide in rats with diabetic neuropathic pain caused no antihyperalgesic effect and induced diastolic hypotension [12]. Continuous infusion of i.t. ziconotide (1 µg/h) in dogs did not affect blood pressure, but i.v. bolus injection (0.1 mg/kg) resulted in a profound fall in mean, diastolic and systolic pressures, accompanied by a corresponding tachycardia and decrease in the respiratory rate [39]. In conscious rats, i.v. administration of ω-conotoxin MVIIA decreased blood pressure in a dose-dependent manner [40], and in rabbits, i.v. ω-MVIIA (100 mg kg^-1^) caused a similar decrease in blood pressure and tachycardia that rapidly reached the maximum [41]. In this study, unlike i.v. ω-conotoxin MVIIA (ziconotide), i.v. Phα1β recombinant induced analgesia in rodent models of neuropathic pain, causing negligible cardiac problems, mechanical allodynia, body shaking, serpentine-like tail movements, and behavioral side effects.

The data in the present study suggest the possibility of a non-spinal route for Phα1β recombinant delivery. The analgesic effects of i.v. administration of the recombinant toxin in neuropathic pain models presented efficacy and time-course effect similar to those of i.t. peptide administration [10, 15, 16]. The intravenous administration of recombinant Phα1β reduced the EAE-elicited multiple sclerosis-like symptoms while ziconotide lacked any significant effect when dosed by the i.v. route [42]. Recently, we reported that a single intrathecal injection of native Phα1β reversed all the glial pathological features of the peripheral inflammation. These data reveal for the first time a venom peptide acting on glial structural remodeling *in vivo* [ 43]. On the other hand, intraplantar administration of Phα1β ameliorates acute pain behavior induced by capsaicin [ 44]. Thus, the peptide induces analgesia through different routes of administration. Phα1β inhibition of different subtypes of high-voltage HVCCs [14] and TRPA1 [34],^ ^a broad channel with a well-defined role in inflammation-associated pain, may account for the action of the toxin.

## Conclusion

The results have advanced the clinical trials and development of i.v. recombinant Phα1β for management of persistent pain conditions. This is especially important when spinal-route administration is not possible.

### Abbreviations

CCI: chronic constriction injury; ED_50_: effective dose; i.p.: intraperitoneal; i.t.: intrathecal; i.v.: intravenous; VSCCs: voltage-sensitive calcium channels.
